# A comparative analysis for spatio-temporal spreading patterns of emergency news

**DOI:** 10.1038/s41598-020-76162-7

**Published:** 2020-11-10

**Authors:** Mingjiao Si, Lizhen Cui, Wei Guo, Qingzhong Li, Lei Liu, Xudong Lu, Xin Lu

**Affiliations:** 1 School of Software, Shandong University, Jinan, 250101 China; 2 Joint SDU-NTU Centre for Artificial Intelligence Research (C-FAIR), Jinan, 250101 China; 3 College of Systems Engineering, National University of Defense Technology, Changsha, 410073 China

**Keywords:** Psychology and behaviour, Natural hazards, Human behaviour

## Abstract

Understanding the propagation characteristics of online emergency news communication is of great importance to guiding emergency management and supporting the dissemination of vital information. However, existing methods are limited to the analysis of the dissemination of online information pertaining to a specific disaster event. To study the quantification of the general spreading patterns and unique dynamic evolution of emergency-related information, we build a systematic, comprehensive evaluation framework and apply it to 81 million reposts from Sina Weibo, Chinese largest online microblogging platform, and perform a comparative analysis with four other types of online information (political, social, techs, and entertainment news). We find that the spreading of emergency news generally exhibits a shorter life cycle, a shorter active period, and fewer fluctuations in the aftermath of the peak than other types of news, while propagation is limited to a few steps from the source. Furthermore, compared with other types of news, fewer users tend to repost the same piece of news multiple times, while user influence (which depends on the number of fans) has the least impact on the number of reposts for news of emergencies. These comparative results provide insights that will be useful in the context of disaster relief, emergency management, and other communication path prediction applications.

## Introduction

Earthquakes, tsunamis, traffic accidents and other emergencies have a huge impact on the economic and social development of all countries in the world every year. An “emergency” is a situation which poses an immediate risk to people’s health, life, property, or the environment^1^. In 2018, 315 large-scale natural disaster events (i.e., those which necessitated requests for external assistance at the national or international level) were recorded in the Emergency Events Database (EM-DAT) with 11,804 deaths and over 68 million people affected across the world at a total cost of $131.7 billion^2^. When emergencies occur, only a small amount of real-time information is available; nevertheless, those directly affected need to understand both what has happened and what is still happening inside and outside the affected area.

Communications networks play a crucial role in achieving this goal, as rescue and recovery depend in large part on the availability of reliable and sustained communications^3^. When emergencies occur, the government is expected to provide information through various media channels to ensure that the public is well-informed. Governments and relevant media sources must be aware of one another’s actions so they can convey emergency information and decisions to the public in the shortest possible time. Some studies have used mobile phone location data to analyze the flow of people in order to guide disaster relief efforts^4–6^. However, conventional communication networks often become fragile after a disaster takes place. Many studies^7–9^ have suggested that social media can help people obtain information and provide useful communication channels during emergencies. Twitter, in particular, has been used to offer a clearer picture of what is currently happening or has happened at the scene of a disaster, even when most other communication networks are no longer available. Moreover, the open and timely circulation of emergency event information is vital for many crucial services, such as event detection^10,11^, disaster prediction and warning^12^, emergency response^13,14^, the evaluation and ranking of risk severity, resource controls and reaction planning^15^, reporting and monitoring of risk performance, and reviewing risk management^16^ frameworks.

Therefore, investigating and understanding the patterns of emergency news dissemination through social media channels is a topic of interest. It can help to not only quickly monitor personnel and efficiently understand the development of public opinion in current emergencies, but also prevent public opinion from getting out of control. However, to explore the information transmission mode of emergencies is facing new challenges. With the development of mobile networks, cloud computing, and big data technology applications, more and more users can get information about emergencies from other individuals and express their opinions to others on the social media platform. The dissemination patterns of emergency information is going to evolve into new trends and imply new spreading patterns. Widely published emergency news stories are typically produced when a microblog is repeatedly retweeted by a large number of media outlets and users in a short period of time. The forwarding of information often has a significant impact on the event and may even trigger secondary emergencies if not executed properly. The advancement of Internet technology has provided a platform for the public to freely express opinions and has promoted the triggering of public opinion crises.

A number of existing studies have investigated the use of social media data and how it can help when emergency events occur. However, those studies did not pay attention to the dissemination characteristics of emergency news. Some have explored the ways of identifying the needs of affected communities^17,18^. For example, Kim and Hastak^19^ identified ways of connecting the affected community with external organizations through online social media platforms (in this case, Facebook) in order to improve the accuracy of emergency information disseminated during floods. Disaster recovery is also a key research topic. Paige^20^ developed Disaster Maps using Facebook usage data in areas impacted by natural hazards, producing arregate pictures of how the population is affected by and responding to the hazard.

An increasing number of researchers have begun to focus on the problem of information transmission in online social networks^21–26^. Lu et al.^27^ investigated social network dynamics and the formation and evolution of online communities for understanding how people responded to the 2011 Japanese earthquake and tsunami using datasets uploaded to Twitter shortly before and after the earthquake^28–30^. Yan et al.^28^ concluded four major purposes for users to visit the microblogging system during a disaster: to seek or provide situation updates, opinions, emotional support, and calls to action. Wang et al.^30^ analyzed tweets posted during Hurricane Sandy (October 22 to November 6, 2012) to study responses from different organizations and concluded that news outlets generated a more significant number of impressions and tweeted more frequently than other organizations. The main aim of those studies is to analyze the changes in users’ emotions, the behaviors of users, and the differences in user responses. When emergencies occur, the relevant personnel and departments in charge of monitoring the situation will likely concern themselves with the following issues: How long will the emergency information dissemination cycle last? How much time passes between the formation of public opinion, the outbreak, and the recovery? Will public opinion continue to outburst once the emergency deescalates? Could accurate information be urgently communicated to the public while false news about an emergency spreads simultaneously? Current studies have not solved these problems.

In addition, most studies about the dissemination of information have been limited to specific emergency events. However, the generalizability of conclusions based on separate events is limited, as their results may only reflect a single specific scenario. It is essential to propose a frame of reasonable and effective measurements to automatically quantify the diffusion patterns of emergency news. Information dissemination and public opinion must be quantitatively analyzed to identify early warnings that can be used for prediction in future scenarios. In addition, diffusion patterns can be used not only to predict new information dissemination trends but also to determine the veracity of rumors^31^. Meanwhile, it is essential to design a comparative experiment^32,33^ through which a sufficient number of different event types can be compared. For this purpose, we aim to design a comprehensive measures for mining the general characteristics of the online transmission of emergencies along with the ways in which the online transmission of information related to emergencies is different from the dissemination of other types of event information.

In this paper, we have formulated a set of comprehensive measures for use in quantifying the effect of microblog news communication in terms of five key aspects: temporal characteristics, topological characteristics, user engagement, user influence and information coverage. Temporal characteristics of emergency news were defined according to the four measurements of life cycle, active period, fluctuation, and inter-person diffusion time. Topological characteristics reflected the range of information dissemination, including size, breadth, and depth. We also proposed the measurement of dissemination efficiency to quantify users’ engagement during the process of information spread. We applied these measurements to a dataset obtained from Sina Weibo and compared the methods of communicating emergency news with the other four kinds of news: political, social, technological, and entertainment news. We also go on to discuss the relationship between the number of news reports and the number of people reporting on the event; consequently, we establish establish a model for automatically calculating information coverage based on the entire set of features used in this paper. Finally, we summarize the unique spreading pattern of emergency news and provide suggestions for emergency decision-makers on how to provide necessary information to more people.

## Data and methods

### Data

#### Data collection

All data used in the present research were retrieved from Sina Weibo, which is the first and largest portal site to provide microblogging services in mainland China. The number of active monthly users of Sina Weibo exceeded the number of Twitter users^34^ in the first quarter of 2017 and increased to 497 million by September 2019. We collected 193 events from the official annual events site^35^, which listed “hot” events on the platform in 2018, including the Annual Hot List and the Monthly Hot List.

**Table 1:** Data description and basic statistics.

Event type	Average reposts	Average comments	Average likes	Average users	Events
Emergency	13,126	8879	38,178	6957	35
Political	12,367	10,094	54,908	9547	36
Social	44,172	37,876	116,743	24,678	30
Tech	172,238	16,299	103,858	30,686	30
Entertainment	236,213	127,568	402,266	20,429	62

Subsequently, we identified corresponding microblogs on Sina Weibo by searching keywords pertaining to these events. We collected the microblogs published by the media accounts which first posted about these topics, since we regarded these as the most representative, and crawled the repost records of these microblogs on Sina Weibo.

The dataset contained detailed forwarding records for 193 popular microblogs, including 81,606,262 forwarding records of 3,514,660 users. For each microblog, we extracted three specific fields: original microblog information, volume list, and repost list. Original microblog information refers to user-generated content published by the original publisher, including user ID, post time, and post content. Volume list references the reposts, comments, and likes of a microblog. (Note that reposts indicates how many times the microblog was reposted rather than how many reposters forwarded said microblog.) On Sina Weibo, users are allowed to repost a microblog multiple times. The ‘Repost list’ shows all forwarding records of a microblog, and each record contains detailed information, including the reposter’s name, repost time, repost content, parent user’s name, and reposts. The ‘Parent user’s name’ record cites users that had microblogs forwarded, which illuminates the forwarding relationships between users. All information above can be used to build a network of reposts. We also collected the number of fans and followers of all users who participated in the reposting in order to investigate whether the users who participated in different types of news reposting had different types of influence.

#### Data processing

In order to study the unique transmission mode of emergency news, we manually classified these news posts into five categories: emergency, entertainment, political, social, and tech news, based on the general news categories used by traditional newspapers, radio, and television media^36^. Each category contained more than 30 events (Table 1). The “emergency” category included natural disasters and accidents which occurred suddenly and caused social chaos as well as public health events which occurred suddenly and likely caused social harm or negative consequences for public health. The “political” category mainly refers to the news posted by public security offices, the procuratorate, the courts, or the Ministry of Justice of the People’s Republic of China (MOJ). “Social” news refers to news items provided by readers, listeners, and audiences, while “entertainment” news refers to news related to film, TV, and music, especially that released by celebrities and public figures. Finally, “techs” news mainly refers to news covering scientific and technological activities and achievements.

**Figure 1:**
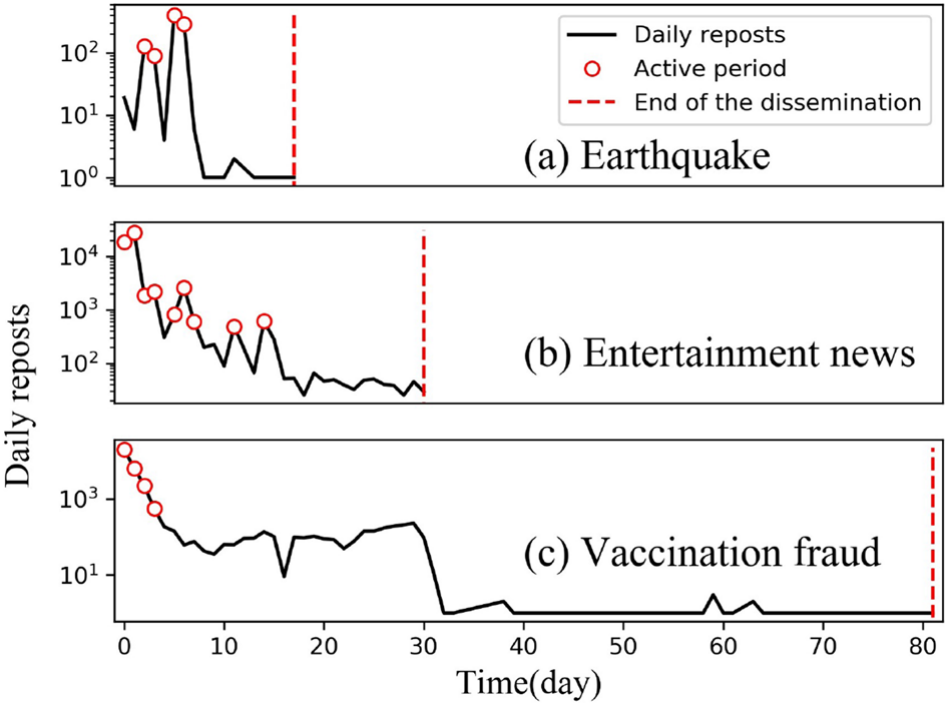
Illustration on the popularity of news over time. (**a**) News about earthquake fluctuation; (**b**) entertainment news typically has a short lifecycle but a long active period; and (**c**) health news often has a long lifecycle but a short active period.

### Temporal measures

In the process of microblog spreading, the popularity of an individual microblog changes over time. Some temporal features may be common for news events of the same type, so we are also interested in whether microblogs belonging to different types of news will have different temporal features.

We tracked the repost records of all events in order to explore the differences in temporal characteristics among different types of events. The popularity of different types of news exhibits unique changes and development trends over time. To visually reflect these changes in terms of the popularity of microblog reposting, a trend chart of repost popularity was drawn for each microblog based on the daily reposts obtained by summing all repost records obtained over 24 h (see Fig. 1 for examples). In order to extract and classify the temporal popularity patterns of reposts, we adopted four measures to quantify the regularity and variety of each event: life cycle, active period, fluctuation, and inter-person diffusion time.

**Life cycle**: The life cycle of an event refers to the entire propagation period of a microblog. A life cycle begins when the microblog is posted by the first user and ends when the last user reposts it. The life cycle $T_l$ of a microblog can be defined as follows:

1 $$\begin{aligned} \left\{ \begin{array}{l}{T_{l}=T_{end}-T_{start}} \\ {T_{\mathrm {start}}=\min \{\mathrm {t} | \mathrm {t} \in \mathrm {T}\}}\\ {T_{\mathrm {end}}=\max \{\mathrm {t} | \mathrm {t} \in \mathrm {T}\}}\end{array}\right. \end{aligned}$$

where $T_{start}$ and $T_{end}$ denote the start time and end time of the entire life cycle, respectively, while *T* is the set of repost times in the repost network *G*. There are significant differences between the life cycles of different microblogs. For example, the life cycle of the vaccination fraud event in Fig. 1c extends over 80 days, while the life cycle of the earthquake event in Fig. 1a is limited to only 15 days.

**Active period**: An ‘active period’ is a measure of how long a microblog maintains high popularity during its life cycle. When considering the entire life cycle, it can be observed that reposts of a specific microblog do not always remain at a high level; it may be heavily reposted only on a few specific days. According to our dataset, more than 95% of microblogs achieve more than 80% of reposts within 30% of their life cycle, which means that the Pareto principle in general applies to the active period of most microblogs.

Thus, the active period is the time during the event when the post experiences its peak popularity. $R = \{r_1,r_2,\ldots ,r_n\}$ is the series of daily reposts for a microblog, where $r_i$ is the number of reposts of day *i* . $\exists R_a \subseteq R$, s.t.$\sum _{r \in R_{a}} r \ge 0.8 \sum _{r \in R} r$, $R_{a}^{*}$ is the collection with the fewest elements, i.e. $\left| R_{a}^{*}\right| \le \left| R_{a}\right|$, $T_{a}=\left| R_{a}^{*}\right|$ is the duration of active period.

**Fluctuation**: To measure the fluctuation of time series of daily reposts, we adopted a method of detecting change point analysis. The Pettitt test is a non-parametric technique for detecting and quantifying significant change points in a time series. Fluctuation is defined as the number of significant change points detected via Pettitt test^37,38^ in the repost time series. A repost time series of a microblog is described as : $X = \{x_1, x_2, x_3, ......, x_T\}$. The most probable change point $\tau$ satisfies:

2 $$\begin{aligned} \begin{aligned} K_{\tau }&= \left| W_{t, T}\right| =\max \left| W_{t, T}\right| \\ W_{t, T}&=\sum _{i=1}^{t} \sum _{j=t+1}^{T} {\varphi }\left( x_{j}-x_{i}\right) , \quad 1 \le t<T, \\ {\varphi }(x_{j}-x_{i})&=\left\{ \begin{array}{ll} -1, &{} \quad \text {if } \left( x_{j}-x_{i}\right) < 0 \\ 0, &{} \quad \text {if } \left( x_{j}-x_{i}\right) = 0 \\ 1, &{} \quad \text {if } \left( x_{j}-x_{i}\right) >0 \\ \end{array}\right. \end{aligned} \end{aligned}$$

Here, $W_{t,T}$ is a statistical index. The signficance probability *p* is evaluated as:

3 $$\begin{aligned} p=2 \exp \left( \frac{-6 K_{\tau }^{2}}{T^{2}+T^{3}}\right) . \end{aligned}$$

$x_\tau$ is a significant change point when $p \le 0.05$. Then, the time series could be divided into two series by $x_\tau$: $X_1 = \{x_1, x_2, x_3, ......, x_\tau \}$; $X_2 = \{ x_{\tau +1}, x_{\tau +2}, ......, x_T\}$. Then, multiple significant change points can be detected by repeating the test for subseries $X_1$ and $X_2$.

**Inter-person diffusion time**: “Inter-person diffusion time” is to measure the period from when the users receive information to when users repost the information^39^. For any microblog *m*, transmission path set $P_m = \{(u_1 , t_1),(u_2 , t_2),\ldots ,(u_k , t_k)\}$, where $t_i$ is the time that user $u_i$ reposted the microblog *m*. If user $u_i$ is a fan of user $u_j$, and $t_j \le t_i$. According to influence timeset $T_i$, inter-person diffusion time $T_p$ of user $u_i$ for microblog *m* is defined as:

4 $$\begin{aligned} \begin{aligned} T_p&= t_i - max(T_i')\\ T_i'&= \{t_j|(u_i,t_i),(u_j,t_j) \in P_m; \{u_i,u_j\} \in E; t_j \le t_i\}. \end{aligned} \end{aligned}$$

### Network topology measures

Different kinds of news spread differently via massive social network. To enable visual observation of these differences, we build a spatial propagation tree for each microblog; this tree visually depicts the spatial distribution of each microblog along with the scope of information coverage at the micro-level.

#### Construction of propagation tree

A propagation tree is modeled as the basis for quantifying the topology of the microblogs’ communication effect. Let $H=({\widetilde{V}}, {\widetilde{E}})$ be an unweighted directed graph that represents a propagation tree for a specific microblog, where ${\tilde{V}}=\left\{ u_{1}, u_{2}, \ldots u_{n}\right\}$ is a node set with *n* users, while ${\widetilde{E}}=\left\{ {\widetilde{E}}_{1}, {\widetilde{E}}_{2}, \ldots {{\widetilde{E}}}_{n}\right\}$ represents a repost relationship set for *n* users. ${\widetilde{E}}_{i}=\left\{ e_{i 1}, e_{i 2}, \ldots e_{i n}\right\}$ is the repost relationship set for user *i*, and $e_{i j}$ is a directional edge between $u_{i}$ and $u_{j}$ that represents the first time at which user *j* reposts a post directly from user *i*. Hence, *H* is a spatial propagation tree for each microblog, where the information source is the root and the forwarding users are the descendant nodes.

#### Topology measures

To understand the general characteristics of each kind of event, we also adopted three structural measures based on the spatial propagation trees, as follows:

**Depth**: The depth *D* of a propagation tree refers to the maximum depth of nodes in the propagation tree:

5 $$\begin{aligned} D=\max \left( d_{i}\right) , i \in [0, n], \end{aligned}$$

where $d_i$ denotes the depth of node *i*, referring to the number of edges from the node to the root node.

**Size**: The size *S* of a propagation tree is the number of unique users who reposted the microblog during its life cycle.

6 $$\begin{aligned} S = \left| U\right| , U = {\{u_{i} |i \in [0,n] \}}. \end{aligned}$$

Here, $u_i$ is user *i* in a propagation tree, while *U* is a set containing all users of this propagation tree.

**Breadth**: The breadth of a propagation tree is a function of its depth. At each depth, the breadth of this tree $b_{j}$ indicates the number of nodes at depth *j*. For a tree with depth *D*, the max-breadth *B* is defined as follows:

7 $$\begin{aligned} B=\max \left( b_{i}\right) , j \in [0, D]. \end{aligned}$$

**Figure 2:**
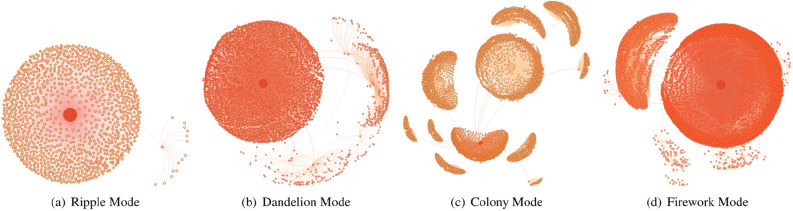
Four spatial propagation modes. Each propagation mode is a directed graph drawn based on the forwarding relationship. The root node represents the original publisher, while the descendant nodes represent the users who engage in forwarding behavior, and the edges represent the forwarding relationships. The color of the node indicates the distance between the source node and the node itself. The closer the source node is, the darker the node’s color; the size of the node is proportional to the degree of each node.

#### Propagation mode

Most propagation trees display similar modes of propagation^40,41^. We visualized the spatial propagation trees of microblogs and classified them into four propagation modes based on their spatial morphology: ripple mode, dandelion mode, colony mode, and firework mode. Different modes imply different degrees of transmission capacity.

**Ripple Mode**: Similar to a ripple spreading outward through water from a center point, ripple mode (Fig. 2a) indicates that the original microblog is directly forwarded from the original publisher. Most user nodes are concentrated around microblog publishers and form a structure that resembles a wave in water. The ripple mode is the most common spreading pattern among all propagation trees. In this mode, messages are typically only reposted by fans of the original publisher.

**Dandelion Mode**: In dandelion mode (Fig. 2b), some users indirectly forward microblogs based on secondary forwarding. This mode exhibits some small breakout points and a weak ability to disseminate information. These nodes can extend the spread of information further than the ripple mode, meaning that many people who are not fans of the original microblog’s publisher can also receive the microblog. However, these nodes also have limited dissemination ability; accordingly, information can usually only be transmitted to fans of these nodes and cannot be passed on to more users.

**Colony Mode**: Colony mode (Fig. 2c) is a specific pattern that contains a limited number of small ripple propagation patterns. In this mode, the root node is not the node with the highest dissemination ability, and many other nodes have reliable dissemination abilities, which enables the colony mode to transmit information to a broader audience. Colony mode is often characterized by a more powerful vitality.

**Firework Mode**: Firework mode (Fig. 2d) has a strong dissemination ability, with many key nodes in the secondary and multiple reposts. This mode combines the characteristics of the dandelion mode (with strong source node transmission) and the colony (with many nodes in the second level of the spatial tree characterized by strong forwarding). However, the nodes in the second layer spread the information more widely compared with those in dandelion mode, and compared with the colony model; moreover, the root node spreads information more widely. These characteristics mean that nodes in firework mode can often spread information to more users who do not know each other. We classified the propagation modes manually and developed a classification method to classify these propagation paths automatically for efficiency. First, we performed comparative experiments using multiple spatial features such as the depth of the propagation tree, the breadth of each layer, and the size of the propagation tree; then we built a logistic regression classification model. We found that breadth sequence was a better performing feature than other features, so we designed our classification model to classify the breadths in all depths of the propagation tree. Then, we design an algorithm based on binary search to infer the two unfixed parameter vector $\gamma = \{\gamma _1, \gamma _2, ... \gamma _9\}$ and $\Gamma = \{\Gamma _1, \Gamma _2, ... \Gamma _9\}$ of this model.

8 $$\begin{aligned} \gamma _{i} \cdot \sum _{j=0}^{h} b_{j}<b_{i}<\Gamma _{i} \cdot \sum _{j=0}^{h} b_{j}, i \in [1,9]. \end{aligned}$$

Here, $b_{j}$ denotes the breadth of a spatial propagation tree at depth *j*, and *h* is the depth of this propagation tree. We fit different values of parameter vectors $\gamma$ and $\Gamma$ for different propagation modes. In the classification of each mode, $\gamma _i$ and $\Gamma _i$ are the threshold values responsible for limiting the number of nodes at level *i*. This model classifies propagation trees by extracting the first nine layers of their features in various propagation modes. We designed an algorithm capable of inferring the undetermined parameters $\gamma$ and $\Gamma$ of the model (see Algorithm 1). Here, input data $B_{i}$ is a list of $b_{i}$ for all propagation trees, *p* denotes the confidence level, and *a* is the minimum interval length that indicates a termination condition for the algorithm.


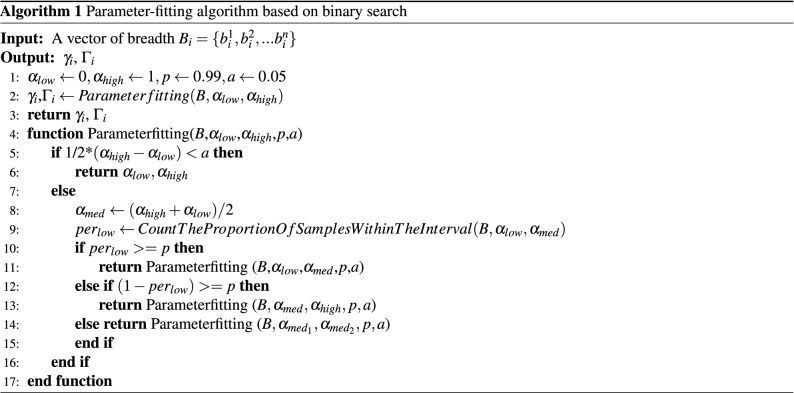


We used the model with its fitted parameters to classify the spatial trees. The accuracy of this model’s classification results and the manual classification results reached more than 96.8%, which is significantly better than the 88.9% achieved by logistic regression models using the feature set with topological measures, as shown in Table 2.

### User engagement measure

The number of times a microblog is reposted is the standard metric used to measure a microblog’s popularity, but this cannot represent the actual number of users involved during the life cycle of an event. Since multiple reposts by one user are permitted on Sina Weibo, the number of reposts on social network sites is very different from the number of people participating in the forwarding. Accordingly, the concept of “efficiency” is proposed to measure user engagement based on the number of reposts and the number of people who actually participated in the forwarding. The efficiency *F* of a type of event is defined as follows:

9 $$\begin{aligned} F= \sum _{n_{i} \in N, r_{i} \in R} \xi \cdot \frac{ n_{i}}{ r_{i}}. \end{aligned}$$

Here, *N* is the set of the number of users who participated in disseminating news related to this type of event, *R* is the set of reposts for this type of event, $n_i$ is the number of users for event *i*, and $r_i$ is the number of reposts of event *i*. Finally, $\xi$ is a weight for the specific event, which is inversely proportional to the number of events in this type of news. The higher the proportion of *F* value, the more users actually participated in forwarding activities and the lower the redundancy of forwarding behavior.

**Table 2:** Performance comparison for the propagation mode classification models.

Feature	Model	Accuracy	Feature	Model	Accuracy
depth + size	logistic	0.819875776	breadth	logistic	0.888198758
breadth + size	logistic	0.869565217	depth	logistic	0.52173913
breadth + depth + size	logistic	0.869565217	size	logistic	0.819875776
breadth + depth	logistic	0.888198758	**breadth**	**our model**	**0.968421053**

### Information coverage measure

In the information dissemination process, it is necessary to spread information to more people effectively. It is generally acknowledged that the larger the number of people who are disseminating information, the more people will receive this information. The repost is usually used as a direct indicator of the number of people who will receive (are “covered by”) the information; however, it can only reflect the number of users who repost the information, not the number of users who actually receive it. On Sina Weibo, when a user posts or reposts a microblog, all of that user’s fans will receive the microblog. Therefore, we define the calculation of the number of users covered in the spread of information as follows:

10 $$\begin{aligned} N_{c} = \sum {F_{i}} , i \in R, \end{aligned}$$

where $N_{c}$ represents the number of covered users, *R* represents reposters, and $F_{i}$ represents the number of fans of reposter *i*.

**Figure 3:**
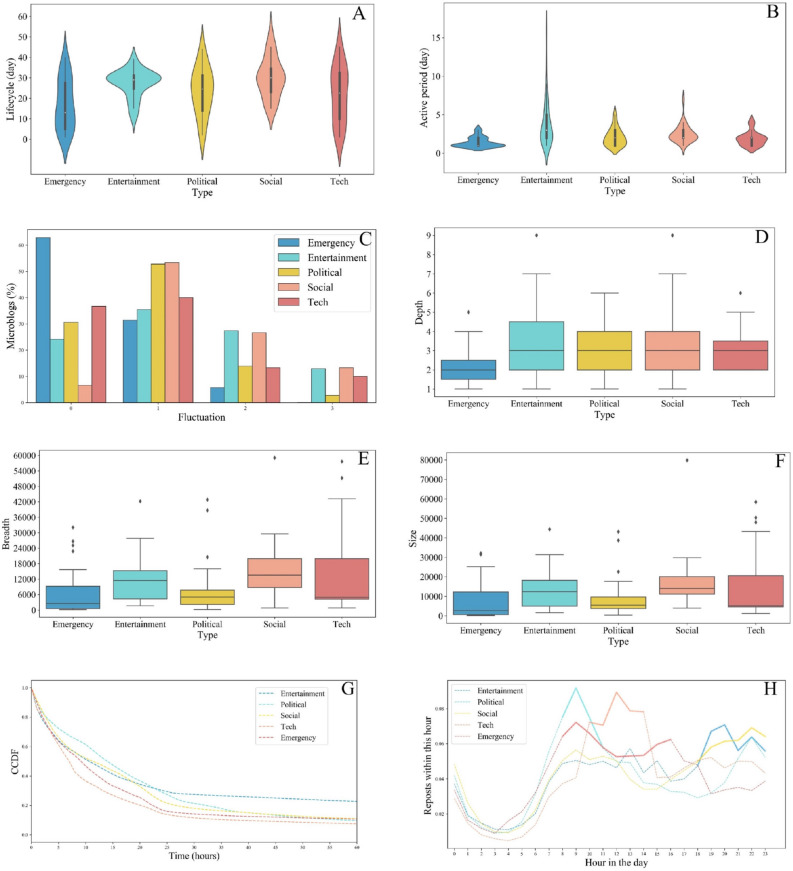
Comparative distribution of the temporal and spatial characteristics of various types of news. (**A**) Violin plot for lifecycle, where lifecycle refers to the length of the entire transmission process. (**B**) Bar plot for active period, where active period refers to the period with the highest number of reposts during the entire transmission process, where $\alpha = 0.2$. (**C**) Bar plot for fluctuation, which shows the percentage of events with more than one fluctuation. (**D**) Box plot for depth, where depth denotes the maximum number of levels from the origin. (**E**) Box plot for breadth, where breadth indicates the maximum number of nodes in the same layer. (**F**) Box plot for size, where size is the number of unique users. (**G**) CCDF (Complementary Cumulative Distribution Function) of inter-person diffusion time, where inter-person diffusion time measures the amount of time between the reposter reposts this microblog and its parent node posts the microblog. (**H**) Distribution of reposts for different types of news according to time of day.

## Results

### Temporal evolution pattern

We begun the investigation by comparing the temporal evolution pattern of emergency news with the other four types of news. To reduce the errors caused by outliers, we used the 5–95 percentiles of each data group.

The life cycle of the dissemination of emergency news ranged from 1 to 45 days, with most emergency events (60.7%) lasting less than 20 days (Fig. 3a). The results for the above three measures indicated that the 15-day median lifecycle of emergency events is the shortest of the five types of events, succeeded by tech news (23 days), entertainment news (29 days), politics (25 days), and social news (30 days). These characteristics illustrate that most emergency news will not be disseminated over a long period of time, as the nature of emergency events means that they last a short period.

The active period of emergency news (Fig. 3b) is concentrated within the initial 1–3 days, with about 93.5% of activity occurring within this period. The mean active period of all microblogs is 1.78, which is far earlier than other types of news (entertainment, 6.75; political, 2.85; tech, 3.18; and social, 5.04). It therefore emerges that users generally did not discuss the same piece of emergency news continuously and passionately; this may be due to emergency news reports, with people soon turning their attention to the latest emergency news reports, which develop rapidly, rather than continuing to discuss previous, outdated reports.

Compared with other types of news, most emergency news stories (over 62.9%) do not experience significant change during the spread period (Fig. 3c). This is much higher than four other news types (entertainment news, 24.3%; social news, 6.7%; techs, 36.7%; and politics, 30.6%). This result indicates that it is rare for the same emergency news story to spike twice, which implies that users typically do not engage enthusiastically with the same piece of emergency news after the active period has passed.

According to the characteristics of all news, political news exhibits similar characteristics to emergency news in three key respects. It is noteworthy that, the propagation mode of entertainment news is different from other types. The active period of entertainment news (Fig. 3b) is generally the longest, often three times longer than that of emergency news. Moreover, its life cycle distribution is relatively clustered. In essence, there are no pieces of entertainment news with very long life cycles, which indicates that the spread of entertainment news is characterized by a short period of intense attention and rapid decay. In addition, the spread of entertainment news tends to undergo more fluctuations than other news types, which indicates that the same piece of entertainment news can be easily re-popularized. Meanwhile, users were likely to come back to express their opinions regarding the same piece of entertainment news after the active period had passed.

The distribution of inter-person diffusion time shows that most reposts occurred less than a day after the parent node was posted. The cumulative distribution demonstrates that the overall inter-person diffusion time of users reposting emergency news and technical news is shorter than that for other events (Fig. 3g).

We also investigated whether users would prefer to repost certain types of news at specific times of day, and our findings are presented in Fig. 3h. We observed that emergency news was reposted most frequently from 8 a.m. to 4 p.m.; political news posts peaked once around 8 a.m. and again around 10 p.m. The noon period, from 10 a.m. to 2 p.m., saw users preferring to forward tech news. The possibility of users forwarding entertainment news and social news gradually increased after 6 p.m.

### Network propagation characteristics

#### Basic characteristics of measures

The maximum propagation depth of all propagation trees was 9 layers (Fig. 3d). This indicates that there were 7 indirect forwarders between the forwarder in the outermost layer and the original publisher. Entertainment news underwent the widest spread, reaching 9 levels, while emergency news exhibited the narrowest spread, within 5 levels. The mean depth of other types of news was 3 levels, while emergency news reached only 2 levels. All other types of news achieved greater levels of dissemination compared to emergency news.

Most max breadths of news ranged from 5000 to 18,000. The spread of social news (Fig. 3e) was the broadest (without considering outliers, the broadest social news event reached 30,000, and the median of social news reached 13,000), while the spread of emergency news was the narrowest (with the most widely spread piece of emergency news reaching only 16,000). Moreover, the median piece of emergency news reached only 1000.

In general, the depth of emergency news was the shallowest (Fig. 3f), which indicates that the number of users who participated in the reposting of emergency news was the smallest. This distribution of depth is consistent with the distribution of breadth, while it is slightly different from the depth distribution of information dissemination.

**Figure 4:**
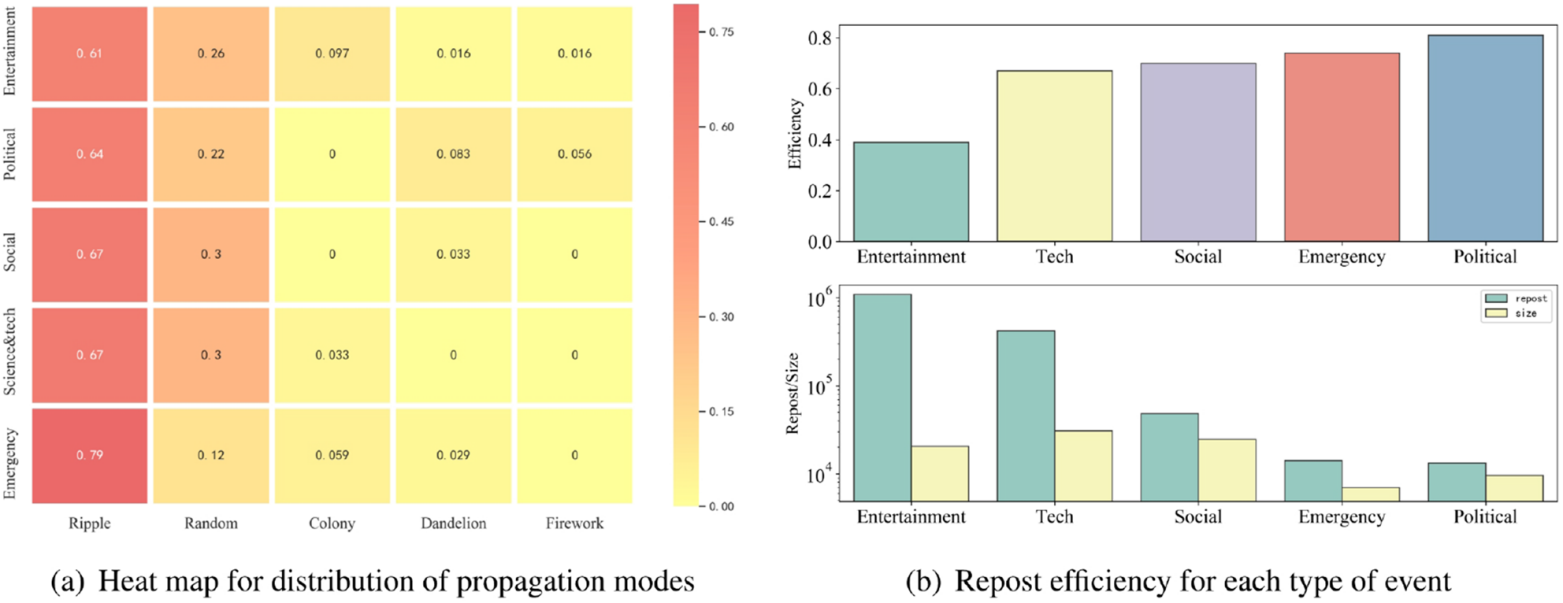
(**a**) Distribution of propagation modes. The number in each cell indicates the relative proportion amongst all news types. (**b**) Number of reposts, unique participating users, and the efficiency for the spread of each type of event. The heat map and the bar were drawn by python 3.7.4 (https://www.python.org/downloads/release/python-374/http://seaborn.pydata.org/index.html), seaborn^42^ libraries in python, version 0.9.0.

#### Classification of propagation modes

We counted the proportion of each mode of propagation for each type of event and illustrated the results using a heat map (Fig. 4a). While the ripple propagation mode was the most common across all news categories, this phenomenon was even more evident in emergency news (where up to 79% of cases were disseminated via ripple mode) compared to other news types. In the emergency news context, few other propagation modes demonstrated durable dissemination ability. Information diffused via ripple mode relies primarily on the popularity of the information source (i.e., the user who first posted the microblog in question). The source node sends the message directly to their fans rather than to other users by means of their fans. Generally speaking, this mode has a relatively small transmission range compared with other modes; this means that emergency news information is typically disseminated to people who are strictly related to the source rather than to a broader audience. In other words, it is often difficult to spread emergency news to a broader range of users. This may be related to the regional nature of emergencies, which are usually concentrated in a geographical area and tend to affect a closely related group of people at the same time.

### User engagement

Emergency news exhibits a higher degree of similarity to political events than other types of events in terms of user participation (Fig. 4b). These two types of news exhibit the fewest reposts but the highest repost efficiency (as high as 70–80%). This indicates that, for emergency news, the number of users who actually participated in disseminating news regarding this event was the closest to the repost value for all types of news; this may be due to the authenticity of political and emergency news.

As can be seen from Fig. 4b, more reposts did not indicate that more users participated in the forwarding; higher numbers of reposts were usually accompanied by lower forwarding efficiency. Entertainment news had the greatest amount of reposts but the lowest efficiency; in other words, the number of users who actually reposted the information was far lower than the repost value. Moreover, this also means that the spread of emergency news likely involved fewer repeated reposts by the same users.

**Figure 5:**
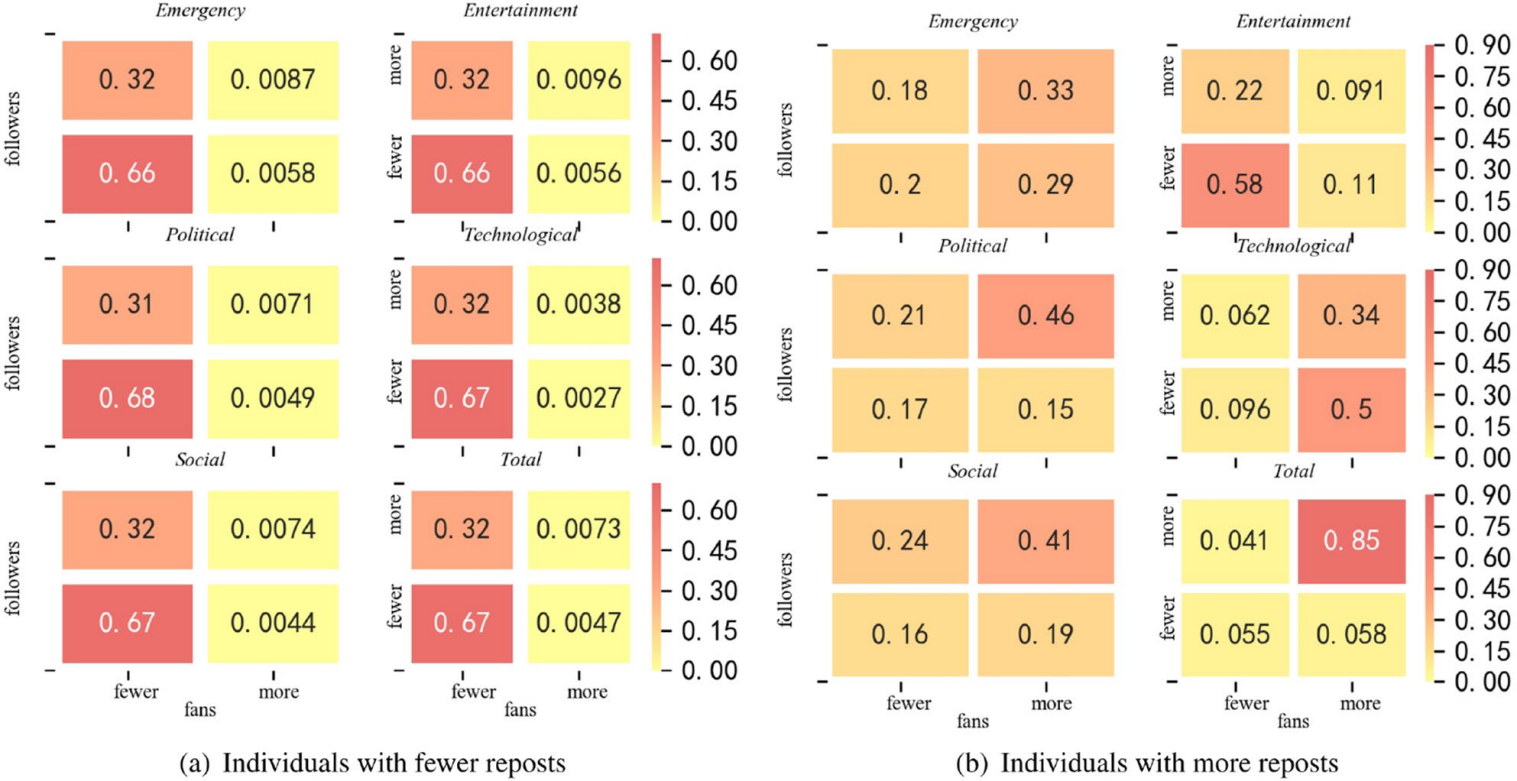
Individual distribution heat map for user influence and reposts. Each number represents a proportion of individuals for each type of news. The charts on the left represent individuals with fewer reposts than the mean, while those on the right indicate individuals with more reposts than the mean. The X axis indicates the number of fans and the Y axis indicates the number of followers. For example, in (**a**) ‘Emergency’, ‘0.32’ means that individuals with fewer fans, more followers, but less reposts account for 32% of the total number of individuals. The heat maps were drawn by python 3.7.4, seaborn^42^ libraries in python, version 0.9.0.

### User influence

User influence is an important factor that impacted information dissemination. To further investigate the relationship between the repost figure for various events and the users’ influence (i.e., the number of fans and followers), we divided the repost-users for each type of event into two categories according to the mean: namely, high-repost users (i.e., those with more reposts) and low-repost users (i.e. those with fewer reposts). Figure 5a illustrates the distribution of low-repost users, and Fig. 5b presents the distribution of high-repost users. We then classified these two types of users according to their number of followers (number of accounts followed by users) and number of fans, respectively, after which we determined their proportion in terms of the number. It was obvious from the chart on the left that the different subcharts were similiar in terms of color distribution, indicating that the distributions of low-repost users are roughly the same for all types of news. However, in the chart on the right, the color distributions of the different subcharts differ significantly, which indicates that the distribution of high-repost users was different for each type of news.

Regarding the distribution of low-repost users (Fig. 5a), the five types of events were similar: 66–69% of these users had fewer followers and fans, while about 32% of these users had more followers and fewer fans. These two groups included the majority of users, which is consistent with the overall population distribution. Less than 1% of users’ reposts exceeded the mean, which indicates that only a few people were message senders, while most people were message receivers. As shown in Fig. 5b, the distribution of high-repost users differs significantly depending on the type of event. In the political and social categories, the distributions were similar; users with more followers and more fans always had a high repost value. This is consistent across all data distributions. However, contrary to expectations, users who had more fans did not always have larger repost values; particularly for entertainment events, many users with fewer fans and less attention tend to have more reposts. Moreover, in the emergency news category (Fig. 5b), among the users with more reposts, there were no obvious differences between the distribution of the number of people at each level in terms of fans or amount of attentions, which can also be inferred from the variance (emergency, 0.0587; entertainment, 0.0641; political, 0.0645; tech, 0.0704; and social, 0.0585). This indicates that the number of reposts does not increase with the number of fans; that is to say, the spread of emergency news is less affected by user influence than other type of news.

**Figure 6:**
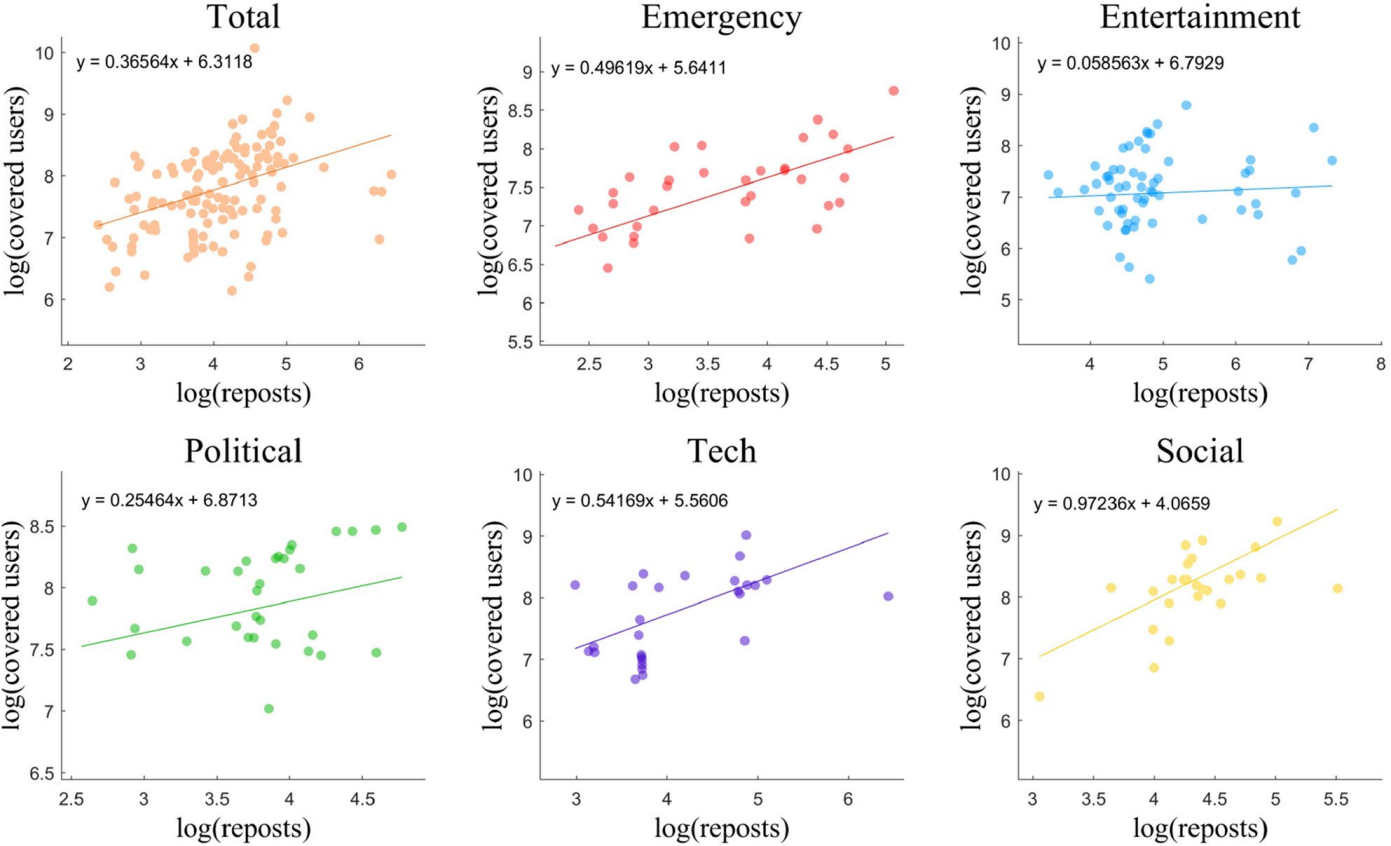
The relationship between repost value and the number of covered users.

### Information coverage

To study the relationship between reposts (number of times forwarded) and the number of users covered by the information, we performed) a linear fitting on the experimental data, as shown in Fig. 6. The result is significant for Emergency, Tech and Social news. According to the significant results of the linear fitting, the larger the intercept, the larger the cardinality of the number of people covered by the information. Moreover, this cardinality can reflect the influence of the original publisher to a certain extent: the larger the intercept, the greater the spread influence of the original publisher. The figures for the three types of news are as follows: ‘social (4.06), tech (5.56), emergency (5.64)’. Generally speaking, the publishers of emergency news had a larger average spread of influence than social and tech new. This was likely because the users who regularly posted emergency news tended to be media accounts with a large number of followers and high attention rates. From the slope of the linear fitting results, moreover, it can be concluded that the larger the slope, the greater the number of people covered per unit of repost when the result is significant. The slopes of the three types of news are as follows: ‘social (0.97), tech (0.54), emergency (0.49)’. Among them, the ranking of social news was ranked highest, which indicates that the number of people who received the information rapidly increased, with increased repost values for this type of information. Moreover, emergency news exhibited the lowest slope ranking, which indicates that the number of people covered by the information in question slowly increased as the repost value increased. As shown in the results of our visualization, the data points for emergencies were the most concentrated around the fitting line, which is conducive to model fitting and prediction. The F-tests results show that the linear fitting of the emergency news was the most significant ($p < 0.0001, F = 21.052$). From the perspective of social network structures, a steep slope indicates that users who forwarded social news had a higher average degree; that is, they had a larger circle of friends and can thus transmit information to more people. The average user who reposted emergency news may have fewer fans than those who posted other types of news. From the analysis of the Sina Weibo forwarding mechanism, moreover, the same user can repeatedly forward the same Weibo; this means that the information coverage does not always increase as reposts increase.

**Table 3:** Comparison of prediction models on information coverage.

Model	MSE	RMSE	MAE	R Squre	AR Squre	PCC (r)	PCC (*p* value)
Linear Regression	9.62E+16	3.10E+08	1.57E+08	0.304075	0.223776	0.596435	8.69E−10
Ridge	9.60E+16	3.10E+08	1.56E+08	0.305497	0.225362	0.596882	8.38E−10
Lasso	9.62E+16	3.10E+08	1.57E+08	0.304075	0.223776	0.596435	8.69E−10
DecisionTree	6.99E+16	2.64E+08	1.34E+08	0.494726	0.436425	0.768885	2.19E−18
RandomForest	5.40E+16	2.32E+08	1.33E+08	0.609683	0.564646	0.78876	7.22E−20
AdaBoost	5.66E+16	2.38E+08	1.27E+08	0.590858	0.543649	0.804804	3.48E−21
GradientBoost	5.86E+16	2.42E+08	1.27E+08	0.576127	0.527218	0.794419	2.56E−20
Bagging	5.52E+16	2.35E+08	1.43E+08	0.60096	0.554917	0.782423	2.23E−19
Our Method	3.20E+16	1.79E+08	1.1E+08	0.76854	0.74183	0.89757	2.46E−32

We used the entire set of features mentioned in this paper to design an experiment for predicting information coverage. We designed an ensemble learning model based on a set of machine learning regression models, including ridge, lasso, DecisionTree, RandomForest, AdaBoost, GradientBoost, and bagging models. The ensemble learning model included *N* component learners $\{h_1,h_2,\ldots ,h_N\}$, where $h_i(x)$ was the output by component learner $h_i$ and *H*(*x*)was the output by the ensemble learning model.

11 $$\begin{aligned} H(x) = \frac{1}{N} \sum _{i = 1}^{N} h_i (x). \end{aligned}$$

This model clearly improved effects compared to other models, as shown in Table 3. Besides, we expand three types of correlations: Pearson correlation, Kendall rank correlation and Spearman correlation. The Spearman and Kendall rank correlation are significant for all features. Result shows that spatial features are most predictive features, and size is the most useful feature to predict the coverage ($$\hbox {r} = 0.54$$, $$ {p} < 0.01$$). While the temporal features ($$\hbox {r} < 0.26$$, $$ {p} < 0.05$$) are not to be indicative of the coverage, especially the life cycle ($$\hbox {r} = 0.14$$, $$ {p} < 0.1$$).

## Conclusions

In this paper, we investigated the unique patterns of the spread of emergency news based on data obtained from Sina Weibo. We put forward quantitative statistics about the propagation rules of other kinds of news to compare and analyze emergency news alongside four different types of news events from four aspects; subsequently, we summarized the discriminant method of the five transmission modes. We found that the spread of emergency news can be described in terms of the following unique patterns: it is transmitted over shorter distances, lasts for a shorter period, and experiences fewer fluctuations.

We found that emergency news usually did not experience a second peak period. Therefore, when the daily reposts of emergency news peaks and then shows a downward trend, we can infer that it will continue to decline. Moreover, we found a unique feature in the propagation of emergency news; in the dissemination of other news, dissemination nodes that generate a large number of reposts usually have a large number of fans, but this is not the case in emergencies. Users with relatively few fans can also get many reposts. Mastering these propagation characteristics can significantly help to predict emergencies and regulate public opinion. At the same time, they can provide some insights and help to predict the dissemination of information. In light of the above information, we recommend that the relevant government departments strengthen the control of emergency news dissemination. First, government departments could intervene when the popularity of emergency news items begins to decline, particularly for news that can provide essential information for emergency management-related work. This method of intervention could cause a second wave of attention to be paid to these news items, thereby extending the news dissemination life cycle.

We further advise that public figures also take an active part in the timely forwarding and dissemination of relevant microblogs, as this will effectively transform the propagation mode of emergencies from a ripple mode to a mode with higher propagation power, such as “‘colony” or “firework” modes. Compared with other types of news, the information sources of emergency news are influential and dominant, so the suppression of fake emergency news dissemination could be well achieved by guiding and controlling the information sources of news dissemination. We also proposed a method to automatically identify information dissemination patterns for the majority of news, and we provided a regression prediction model based on ensemble learning to predict the number of users covered by information. Experiments proved that our models perform well. In future work, we will further work on elaborating the reasons for the propagation characteristics of emergencies and describe how to predict these propagation characteristics. Our future work will also attempt to identify the veracity of news shared on Sina Weibo during different events based on the features defined in this paper.
